# Dentofacial Medicine and Diagnosis

**DOI:** 10.2174/1745017901814010696

**Published:** 2018-09-28

**Authors:** Deepak Gupta

**Affiliations:** Department of Oral Medicine and Radiology M.M. College of Dental Sciences and Research

“Dentofacial medicine and diagnosis” refers to the diagnosis and management of oral diseases. It aims to promote and advance the art and science of oral medicine and diagnosis in dentistry. It includes early diagnosis as well as interpretation of oral manifestations of systemic diseases as well. Since oral health has got a pivotal role for maintaining the overall health of an individual, this special issue highlights the importance of dentofacial medicine for the early diagnosis and treatment planning. However, this particular fact is already known by the general dental practitioners, still it is not a well practiced entity. This leads to increased mortality as well as morbidity. Henceforth this issue is deemed necessary to highlight few of very important aspects including early clinical diagnosis of oral cancer/pre-cancer, oro-facial pain (Including neuralgias), oral lesions and conditions, temporomandibular joint disorders, oral manifestations of systemic diseases, forensic aspect with age estimation, oral psychosomatic disorders *etc*, [1-5]. It is believed that the knowledge of this speciality will reduce the global rate of mortality and morbidly of systemic diseases too as these specialists possess knowledge to diagnose oral signs of these systemic diseases such as Tuberculosis, syphilis, AIDS, Blood cancers, Anaemia, Nutritional diseases *etc*, [1, 4, 5].

In many parts of the world oral medicine and diagnosis is considered to be a major speciality. This is taught to the students as one subject or in collaboration with oral pathology and oral radiology. It is believed that if a dental professional possesses the cutting edge knowledge of oral medicine, it will become easier to diagnose the disease at an earlier stage with better patient compliance [1-3]. As this thematic issue will offer a forum for international collaboration in the field of oral medicine, the coverage will feature cutting-edge research papers, review articles, case reports, diagnostic image reports, technological developments, invited papers, and technical notes. This issue highlights various clinical as well as radiographic expressions and descriptions of various oral and maxillofacial diseases and disorders so as to aid the dental professionals regarding innovative clinical and imaging technique applications.

Following is the brief description of the articles:

## 1 A Randomized, Double-Blind, Placebo-Controlled Trial on Clinical Efficacy of Topical Agents in Reducing Pain and Frequency of Recurrent Aphthous Ulcers.

This paper evaluated the clinical efficacy of various topical agents in order to find the better treatment modality for recurrent apthous stomatitis in terms of reduction in the number, size, exudate level and discomfort associated with pain of these ulcers. Patients were divided into groups and were given 5% Amlexanox, 0.1% Triamcinolone Acetonide, 20% Benzocaine gel, 100 mg Doxycycline hyclate mixed with denture adhesive and normal saline (20:2:1). The study was placebo-controlled in which placebo gel 10gm was used. It was concluded that the topical treatment modalities can deliver cheap, effective and safe drug therapy which benefits the patient in refining their regular activities and everyday events of life.

## 2 Molar Incisor Hypomineralisation: Prevalence and Risk Factors among 7-9 Years Old School Children

The aim of this school-based, cross- sectional survey was to investigate the prevalence and risk factors of permanent Molar incisor hypomineralization among 7-9 years old school children. Molar incisor hypomineralisation were recorded using modified developmental defect of enamel index developed by Clarkson J.J. & O’ Mullane D.M. in 1989and dental caries by using Decayed Missing Filled Tooth index (World Health Organization Modification 1997).It was concluded that molar incisor hypomineralisation was found to affect 2 out of every 10 children examined which was higher than observed in any other study on Indian children.

## 3 Ultrasonographic Characteristics of the Masseter Muscle in Dentate and Edentulous Patients

The purpose of this study was to assess the masseter muscle appearance and thickness while the teeth were relaxed during occlusion and contracted during maximal clenching in fully dentate and in unilateral edentulous patients by ultrasonography. Twenty-five and twenty-four edentulous patients without TMD were enrolled in this study. It was concluded that there were significant differences in the thickness at rest and contraction between the dentate and edentulous groups. It was clarified that ultrasonographic features of the masseter muscle in dentate and edentulous patients were different.

## 4 To Evaluate the Severity, Distribution of Occlusal Tooth Wear and its correlation with Bite force in Young North Indian Adults

The aim of this study was to determine the severity and distribution of occlusal tooth wear among young North Indian adults and to evaluate the correlation of occlusal tooth wear with bite force. A total of 164 subjects were enrolled having full complement of natural dentition (excluding third molars), with no history of orthodontic treatment, FPD and trauma. Maxillary and mandibular casts of each subject were taken. Both maxillary and mandibular anterior and posterior teeth were assessed for severity of wear using a five-point (0 to 4) ordinal scoring system and were then compared with other parameters. It was concluded that tooth wear had significant correlation with age. Further,group function had shown significant occlusal tooth wear as compared to canine guided occlusion.

## 5 Pathogenesis, Clinical Features, Diagnosis and Management of Radiation Hazards in Dentistry

This present article highlights the pathogenesis, clinical features, diagnosis and pharmacological management of radiation hazards with description of minimizing radiation hazards in dentistry. With the advent of newer radiographic diagnostic procedures of the maxillofacial region, there is a drastic increase in the use of Ionizing radiation with a concomitant increase in exposure of patients and health workers to radiation hazards. In addition to the diagnostic information extracted, the radiation exposure may carry the potential for harm by inducing carcino-genesis. However the amount of radiation exposure during dental treatment might be low but patients are exposed to repeated examinations very often. This is further backed by the fact that dentists require radiographic examination very often. Hence it is recommended to ensure minimum and inevitable exposure during dental treatment.

## 6 Assessment of Mandibular Surface Area Changes in Bruxers *versus* Controls on Panoramic Radiographic Images: A Case Control Study.

Bruxism is the commonest of the many parafunctional activities of the masticatory system. Opinions on the causes of bruxism were numerous and widely varying. It can occur on sleep as well as wakefulness. Bruxism was for long considered a major cause of tooth wear. In this study the area change as measured from digital panoramic radiographs that can occur on the lower jaw bone in those with Bruxism was compared with non bruxers. The results of this study reveals that the condylar and coronoid process in such patients show significant changes.

## 7 Digitized Morphometric Analysis Using Maxillary Canine and Mandibular First Molar for Age Estimation in South Indian Population

The aim of this study was to derive precise population specific formulae for age estimation. Digitalized orthopantomography of 150 subjects, was retrieved. The subjects were divided into study and test group. No significant difference between mean chronological age and mean estimated age were found. It was concluded that the procedure using anterior and posterior teeth together gave more accurate results. The prediction accuracy can be further enhanced by using multiple teeth or by utilizing other linear measurements in the same teeth.

## 8 Correlation of Condylar Translation During Maximal Mouth Opening with Presence of Signs of Temporomandibular Joint Disorders in an Asymptomatic Population of 18-25 Years Age Group of Northern India.

The objective of this study was to determine the frequency of “subluxation” and presence of clinical signs of TMD in asymptomatic individuals and its distribution according to age and sex. The material investigated comprised of 200 asymptomatic subjects with 400 joints. The material was divided into two groups of 18-25 years and 50-60 years of age consisting of equal number of males and females. It was concluded that subluxation is a very common feature found in almost all the individuals and its prevalence is significantly very high. Hence it was assumed that the increase incidence of TMDs could be a direct result of the phenomena of subluxation. The decrease in mandibular length could be the cause of decreased mouth opening and increased subluxation.

## 9 Diagnostic accuracy of fine needle aspiration cytology in lesions of oral cavity and salivary glands: A clinico-pathological study.

Fine Needle Aspiration Cytology (FNAC) is a rapid, reliable and safe diagnostic tool used for various lesions of oral cavity and salivary glands. The present study was undertaken to categorize the cytomorphology of oral cavity and salivary gland lesions on FNAC and to assess the accuracy of FNAC in arriving at diagnosis. There were 12 aspirates obtained from oral cavity swellings and 58 aspirates were obtained from salivary glands. It was concluded that FNAC is a safe, cost effective and reliable technique effective in diagnosing the spectrum of different lesions in oral and maxillofacial region.

The editorial board of this special issue of The Open Dentistry Journal features a panel of international expert reviewers who has provided their expertise and guidance in shaping the contents of the issue. Once again I would like to extend my thanks to the Editor in Chief of The Open Dentistry Journal for considering this important special issue to uncover the current state of the art scientific literatures and future direction in the field .
